# Efficacy of heel lifts for mid-portion Achilles tendinopathy (the LIFT trial): study protocol for a randomised controlled trial

**DOI:** 10.1186/s13063-024-08185-8

**Published:** 2024-05-24

**Authors:** Jaryd Bourke, Shannon Munteanu, Alessandro Garofolini, Simon Taylor, Peter Malliaras

**Affiliations:** 1 Physiotherapy Department, School of Primary and Allied Health Care, Faculty of Medicine Nursing and Health Science, Monash University, Victoria, Australia; 2 Discipline of Podiatry, School of Allied Health, Human Services and Sport, La Trobe University, Victoria, Australia; 3 Institute for Health and Sport (IHES), Victoria University, Victoria, Australia

**Keywords:** Tendinopathy, Achilles tendon, Heel lift, Orthotic devices, Clinical trial protocol

## Abstract

**Background:**

Mid-portion Achilles tendinopathy is a common condition, characterised by localised Achilles tendon load-related pain and dysfunction. Numerous non-surgical treatments have been proposed for the treatment of this condition, but many of these treatments have a poor or non-existent evidence base. Heel lifts have also been advocated as a treatment for Achilles tendinopathy, but the efficacy and mechanism of action of this intervention is unclear. This proposal describes a randomised controlled trial comparing the effectiveness of heel lifts versus sham heel lifts for reducing pain associated with mid-portion Achilles tendinopathy, with an embedded biomechanical analysis.

**Methods:**

One hundred and eight men and women aged 18 to 65 years with mid-portion Achilles tendinopathy (who satisfy the inclusion and exclusion criteria) will be recruited. Participants will be randomised, using the website Sealed Envelope, to either a control group (sham heel lifts) or an experimental group (heel lifts). Both groups will be provided with education regarding acceptable pain levels to ensure all participants receive some form of treatment. The participants will be instructed to use their allocated intervention for at least 8 h every day for 12 weeks. The primary outcome measure will be pain intensity (numerical rating scale) at its worst over the previous week. The secondary outcome measures will be additional measures of Achilles tendon pain and disability, participant-perceived global ratings of change, function, level of physical activity and health-related quality of life. Data will be collected at baseline and the primary endpoint (week 12). Data will be analysed using the intention-to-treat principle. In addition, the acute kinetic and kinematic effects of the interventions will be examined at baseline in a subpopulation of the participants (*n* = 40) while walking and running using three-dimensional motion analysis.

**Discussion:**

The LIFT trial (efficacy of heeL lIfts For mid-portion Achilles Tendinopathy) will be the first randomised trial to compare the efficacy of heel lifts to a sham intervention in reducing pain and disability in people with Achilles tendinopathy. The biomechanical analysis will provide useful insights into the mechanism of action of heel lifts.

**Trial registration:**

Australian New Zealand Clinical Trials Registry, ACTRN12623000627651. Registered 7 June 2023.

**Supplementary Information:**

The online version contains supplementary material available at 10.1186/s13063-024-08185-8.

## Introduction

### Background and rationale

Mid-portion Achilles tendinopathy (AT) is a common condition, with incidence rates reported to be 2.35 per 1000 people in the general population [1]. Athletes, particularly elite long-distance runners, are commonly affected having a lifetime risk of 52% [2]. However, the condition also affects sedentary populations, with one third of people with chronic AT being physically inactive [3]. Achilles tendinopathy typically causes localised load-related pain and stiffness, which can be debilitating and severely impact quality of life [4]. In addition, recovery times can be long (between 21 and 479 days [5]) and treatment expensive; in the Netherlands, the mean estimated costs were US $490 per patient (mostly from healthcare visits) per annum [4].

Achilles tendon loading exercise accompanied with education about limiting pain with activity is recommended as the first-line treatment for AT in clinical guidelines [6, 7] and expert narratives [8, 9]. However, the efficacy of exercise is unclear, as there are major criticisms of current research in this area, including underreporting and underpowered studies [10]. Also, exercise alone may not always be effective as up to 60% of people with AT have continued pain and disability after 5 years despite exercise interventions, and 48% will seek additional treatment including shoe inserts and surgery [11].

Heel lifts are an alternative treatment that are commonly used and recommended for mid-portion AT [12, 13]. A recent trial has supported the use of this intervention for mid-portion AT, reporting that heel lifts were more effective than eccentric calf exercise over 12 weeks for improving pain, function, and health-related quality of life [14]. However, there were limitations to this trial, including (i) participants were not blinded to the allocated intervention and (ii) there was an absence of a no treatment group (e.g. sham). Therefore, it is unclear if the efficacy of the heel lifts was due to specific treatment effects, placebo effects or other factors, such as natural history.

The classical theoretical mechanism for the use of heel lifts is that they reduce the load demands placed on the Achilles tendon (i.e. strain and peak force) by increasing the lever arm of the tendon at the ankle [15]. However, the biomechanical studies on the Achilles tendon and heel lifts thus far have been limited to small trials (i.e. 3 to 19 participants), who were all healthy [16] and may have differing responses compared to a symptomatic AT cohort. Therefore, there is currently a lack of evidence to explain the mechanism by which heel lifts exert their effects when used to treat mid-portion Achilles tendinopathy.

### Objectives

The primary aim is to examine the efficacy of heel lifts versus sham heel lifts on pain intensity among individuals with mid-portion Achilles tendinopathy at 12 weeks. The secondary aim is to investigate the acute biomechanical effects of heel lifts among individuals with mid-portion Achilles tendinopathy.

## Methods

### Trial design

**Fig. 1:**
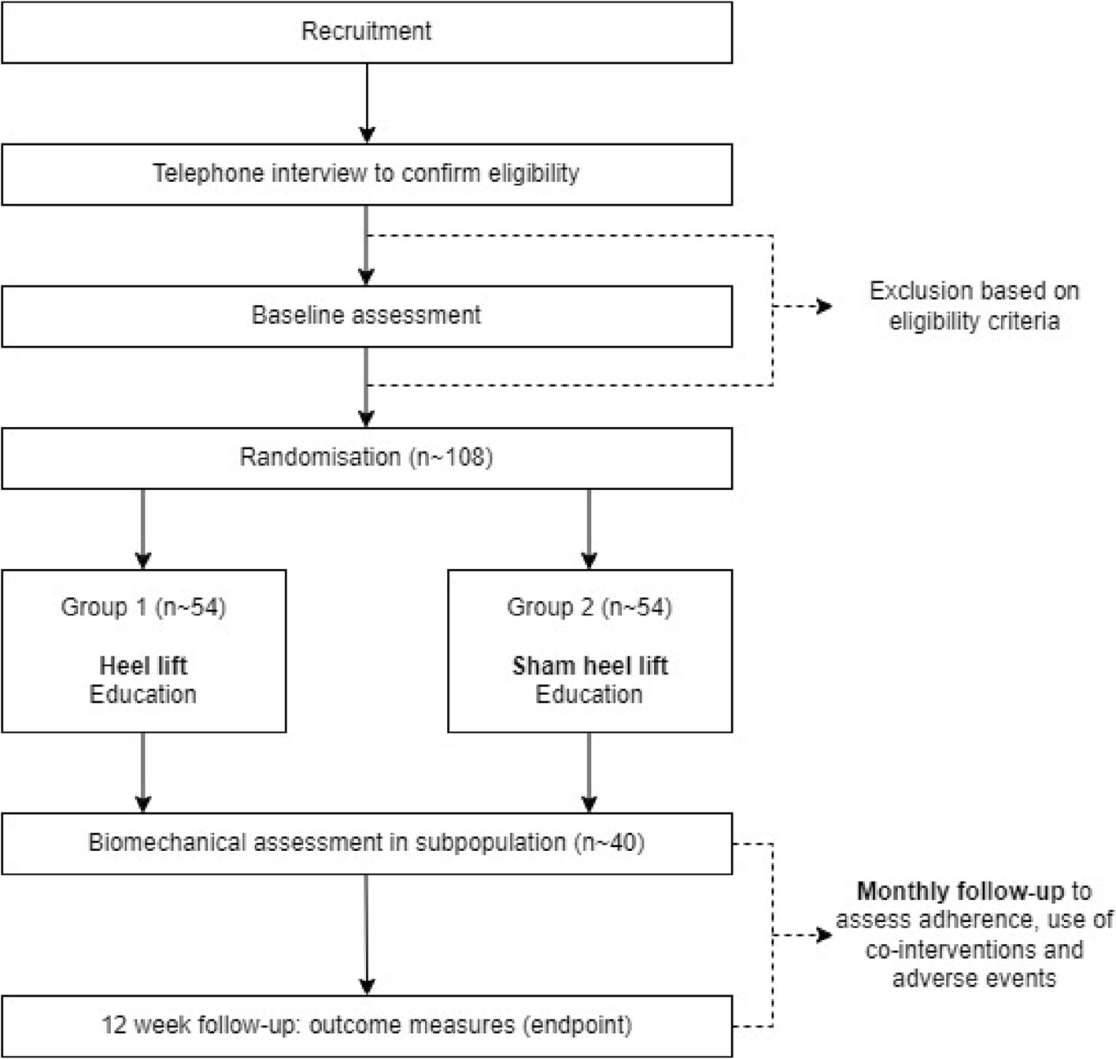
Study protocol

The LIFT trial (efficacy of heeL lIfts For mid-portion Achilles Tendinopathy) will be a parallel group, participant- and assessor-blinded, explanatory, superiority randomised controlled trial with a 12-week follow-up (Fig. 1). Participants will be randomised to a control group (sham heel lift) or an experimental group (heel lifts). To ensure all participants (who will all have some level of pain and disability) receive some form of intervention, both groups will be provided with the same education regarding activity modification. This trial design covers any ethical concerns of not treating participants in pain and will allow the efficacy of heel lifts to be evaluated.

This trial protocol has been reported in accordance with the Standard Protocol Items: Recommendations for Interventional Trials (SPIRIT) guidelines [17], as well as tendinopathy consensus group reporting recommendations [18]. The SPIRIT checklist is included as Additional file 1. Any changes to the trial protocol will be reported in the final publication and communicated to relevant parties, such as the Ethics Committee, trial registry and participants.

### Study setting

The initial assessment will be conducted at a single centre in the Institute for Health and Sport (Victoria University, Melbourne Australia). Publications associated with the trial will be reported according to the Consolidated Standards of Reporting Trials (CONSORT) 2010 [19, 20], and the interventions will be described using the TIDieR checklist [21]. The trial has been registered with the Australian New Zealand Clinical Trials Registry (ACTRN12623000627651).

### Ethics approval

Ethics approval has been granted from the Monash University Human Ethics Committee (no: 36420). Informed consent will be obtained from all participants (Additional File 2). Ethical standards will adhere to the National Health and Medical Research Council (NHMRC) National Statement [22] and the World Medical Association’s Declaration of Helsinki [23].

### Eligibility criteria

Participants will be recruited by mail-out and emailed advertisements to healthcare practitioners’ in Melbourne. In addition, we will advertise this trial using social media (such as Twitter, Facebook and Instagram), including paid advertisements.

All participants who meet the eligibility criteria will be included. Respondents will initially be screened by telephone interview by a single researcher (JB) to check that they are suitable for the study. Suitable participants will then attend an initial assessment for further eligibility screening. The assessing investigator (JB) is a registered podiatrist with 3 years of experience and will be supported by investigators with expertise in Achilles tendinopathy management and clinical trials (PM: registered physiotherapist; SEM: registered podiatrist).

Inclusion criteria:

- Aged 18 to 65 years;
- Symptoms of mid-portion Achilles tendinopathy in one or both lower limb(s) for > 6 weeks;
- Report maximum Achilles tendon pain severity experienced over the past week that is > 3 out of 10 (using a numerical pain rating scale);
- Regularly use footwear that can accommodate heel lifts. This is defined as using footwear that can accommodate heel lifts for at least 8 h per day [24];
- Be literate in English and able to complete the questionnaires used in this trial (e.g. VISA-A questionnaire);
- Be willing to not receive any treatment on the involved Achilles tendon(s) (other than those allocated in the current study) during the study period;
- Be willing and able to attend Victoria University (Melbourne, Australia) on one occasion for assessment.

Mid-portion Achilles tendinopathy will be diagnosed as per the clinical guidelines [7] and musculoskeletal ultrasound [25] using the following criteria:

- Report pain in the Achilles tendon during or after weight-bearing activities including walking, running or jumping/hopping;
- Pain in the Achilles tendon 2–6 cm proximal to the insertion (as described by the patient and palpated by the investigator);
- Gray-scale musculoskeletal ultrasound of the Achilles tendon(s) showing diffuse or local thickening (anterior–posterior) and/or irregular fibre orientation and/or hypoechoic areas within the mid-portion of the Achilles tendon. Certain features are commonly associated with mid-portion Achilles tendinopathy; however, may also exist in asymptomatic individuals [25]. Therefore, if participants exhibit the aforementioned sonographic features accompanied by fluid in the retrocalcaneal bursae (up to 4 mm), focal calcifications, paratenon thickening or calcaneal cortical anomalies (e.g. spurring); they will not be excluded [26].

Exclusion criteria:

- Currently pregnant;
- Previous Achilles tendon rupture or surgery in the symptomatic lower limb;
- Injury or pathology of the lower limb and/or back or any condition that, in the opinion of the investigators, may interfere with participation in the study (e.g. chronic ankle instability);
- Concurrent conditions (ankle or other region) that are more severe (pain numerical rating scale) than their worst mid-portion Achilles tendinopathy pain;
- Treatment with foot orthoses or heel lifts within the previous 3 months;
- Previous breast cancer/and or use of oestrogen inhibitors;
- Inflammatory arthritis (e.g. psoriatic arthritis);
- Neurological disorders (e.g. Charcot–Marie–Tooth disease);
- Taken fluoroquinolone antibiotics within the previous 2 years;
- Any injection (e.g. corticosteroid) into the Achilles tendon or surrounding area in the previous 3 months;
- Any medical condition that deems a participant unsuitable, based on the opinion of the investigators (e.g. type I or II diabetes).

### Baseline assessment

The baseline assessments were derived from the ICON statement [18]. Participant characteristics (such as age, sex, ethnicity (including if identifies as a First Nations person), education and employment status), smoking status, major medical conditions, number of medications and presentation of tendinopathy (unilateral/bilateral and duration) will be recorded via a custom questionnaire. Height and weight will be measured using a stadiometer and digital scales and body mass index will be calculated as weight (kg)/height (m^2^). Static foot posture will be assessed using the Foot Posture Index (FPI) [27]. Ankle dorsiflexion range of motion will be assessed with a reliable lunge test technique [28]. Participants’ footwear will be assessed using selected items from the Footwear Assessment Tool [29] and their shoe size documented.

### Interventions

#### Randomisation and blinding

Participants will be randomised to one of two groups: an intervention group (heel lifts) or a control group (sham heel lifts). Both groups will also receive the same guideline recommended education about activity modification. Using Sealed Envelope (https://www.sealedenvelope.com/), participants will be randomised on a 1:1 ratio with random permuted block sizes. Participants will be blinded via limited disclosure, as they will be told that the study will be comparing two types of ‘shoe inserts’. Primary and secondary outcomes will be self-reported; thus, this trial will be assessor blinded. The clinician (JB) administering the interventions will be unable to be blinded. The instructions on using the shoe inserts and education provided will be detailed in a brochure provided to the participants at baseline (Supplementary Material).

#### Heel lift (intervention group)

**Fig. 2:**
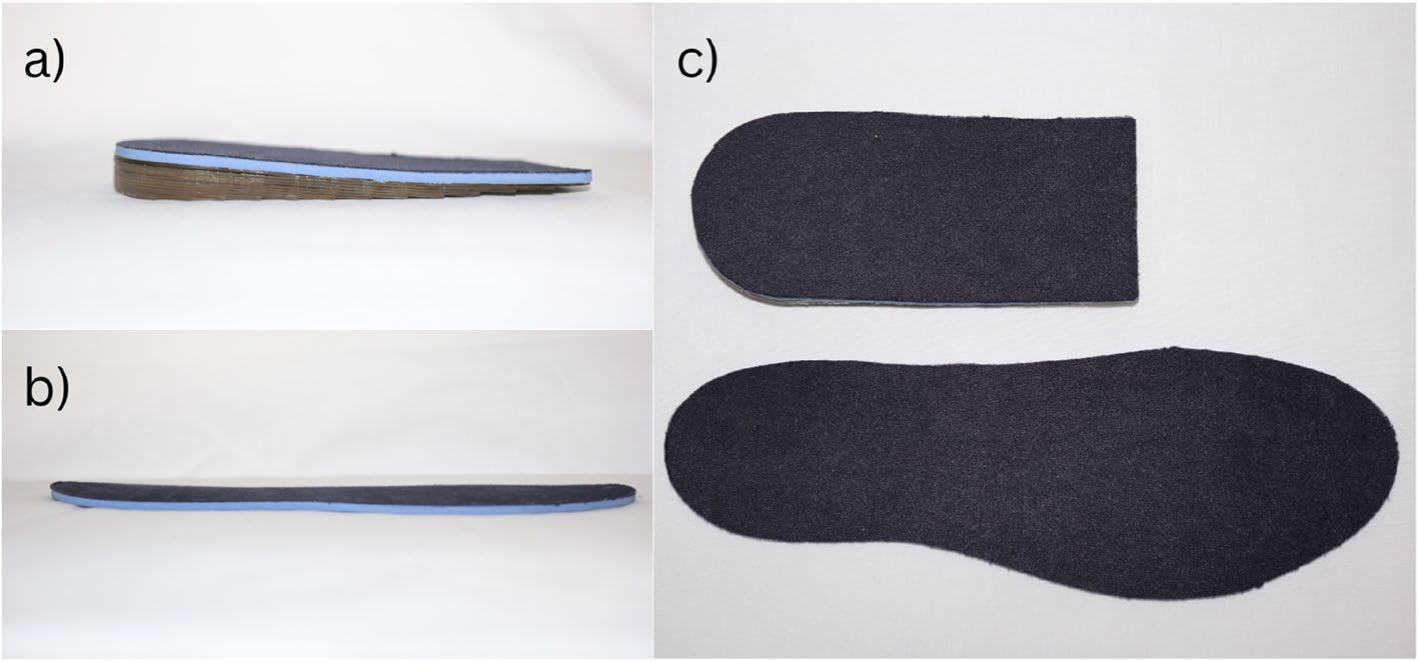
Interventions. **a** Lateral view of heel lift. **b** Lateral view of sham heel lift. **c** Dorsal view of interventions (top: heel lift and bottom: sham heel lift)

Participants allocated to the intervention group will be given a pair of commercially available heel lifts (Clearly Adjustable) for bilateral use. The heel lifts are 12 mm in height and made from firm (Shore A 90) multi-layered clear vinyl. To maximise comfort, a 3.2-mm PPT Ultralux top cover will be adhered to the top surface (Fig. 2). The heel lifts will be reduced in 1-mm increments if required (e.g. heel slippage) and the final height will be recorded at baseline. Small, medium and large heel lifts will be available and issued according to the participant’s shoe size. Participants will be shown how to use the heel lifts and asked to wear them for at least 8 h every day. The decision to use these heel lifts were based on the findings of a previous trial that reported this intervention to be safe and more effective for some clinical outcomes than eccentric calf exercises for mid-portion AT over 12 weeks [14].

#### Sham intervention (control group)

Participants allocated to the control group will receive a pair of the sham heel lifts. The following sham intervention may be used; however, we will pilot other interventions (i.e. made of different materials and dimensions) prior to the trial and reserve the right to change the design if required (this will be reported as a change in the protocol on the website trial registration). The intended design of the sham intervention is to not plantarflex the ankle joint but appear as identical to the heel lifts as possible. To achieve this, the sham heel lift will extend the entire length of the shoe and will be made of the same materials as the heel lift (i.e. 1 mm of clear vinyl with a 3.2-mm PPT Ultralux top cover) (Fig. 2). This sham heel lift is necessary in this trial due to participant’s expectation of receiving a ‘take-home’ intervention (i.e. minimises resentful demoralization [30]). The sham intervention will be sized according to shoe size and trimmed (if needed) to fit into the participant’s footwear. Participants will be shown how to use the sham intervention and asked to wear them for at least 8 h every day.

#### Education

Participants will be given education regarding the amount of acceptable pain during activity, based on the pain-monitoring model [31]. This approach allows individuals with Achilles tendinopathy to continue with some level of physical activity, while being safe and produces comparable outcomes to complete rest from aggravating activities [31]. Participants will be asked to maintain their regular activities/occupations (rather than complete rest) after receiving their allocated ‘shoe insert’, provided the amount of pain they experience in the Achilles tendon pain does not exceed a score of 5 on a 0–10 pain scale, where 0 is no pain and 10 is worst pain imaginable during exercise/activity. The pain after usual physical activities can reach a 5 on the pain scale but should subside by the following morning. During activity, if the pain in the Achilles tendon exceeds 5 on the pain scale, participants will need to reduce their activity/exercise (if possible).

During the trial, participants will be asked to refrain from using any other treatments for their Achilles tendon pain (besides what they are given in this trial). They will be able to take 500 mg of paracetamol on an ad hoc basis if the tendon(s) is painful and asked to document usage. If participants experience any adverse event (e.g. develop new pain) or have concerns (e.g. uncertainty on using their allocated intervention), they will be encouraged to contact a trial investigator (JB, with support from SM and PM), who will decide if it is safe for the participant to continue with their allocated intervention.

### Treatment credibility/expectation

The outcome may be influenced by the participant’s expectations and their beliefs about the credibility of the intervention [32, 33]. Therefore, the credibility of the intervention (participants’ beliefs about the logic underpinning the intervention) and expectancy (participants’ perceptions of how much they may benefit) will be quantified using the Credibility/Expectancy Questionnaire (CEQ) [34]. The CEQ will be administered after the random allocation of the interventions. The CEQ consists of six items that ask participants to rate the credibility of the intervention and their expectancy on a 9-point Likert scale. Higher scores on the scale indicate that the participant considers the intervention to be credible and expects it to be effective. The CEQ is a reliable scale, shown to have good internal consistency and test–retest reliability [34].

### Outcomes

**Fig. 3:**
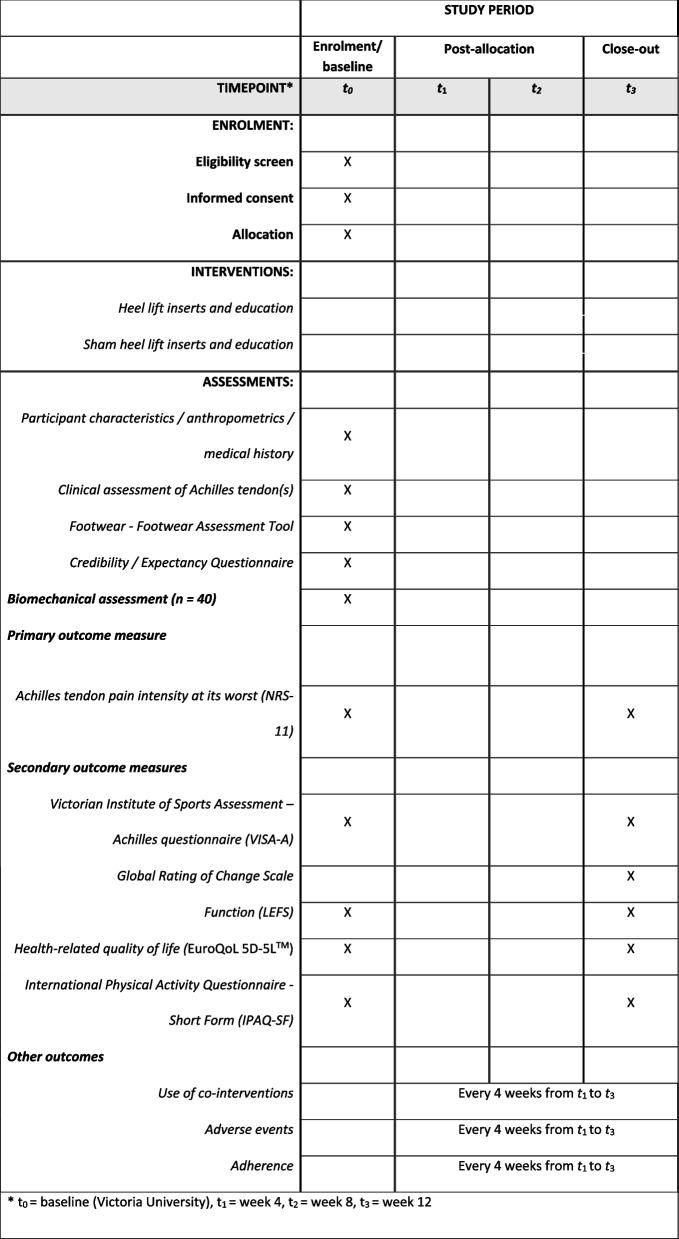
SPIRIT diagram of enrolment, interventions and assessments for the LIFT trial

Primary and secondary outcome measures [35] will be collected at baseline and at 12 weeks (the endpoint). A summary of the data collection time points for each of the outcome measures is shown in Fig. 3. Follow-up data will be collected using REDCap™ surveys; however, participants will have the option of a postal survey. Where participants have bilateral symptoms, they will be asked to report for their most painful side (or right side, if the Achilles tendons are equally painful) to maintain independence of data [36, 37].

#### Primary outcome

The primary outcome will be pain intensity at its worst in the previous week using a 11-point numerical rating scale (NRS) with terminal descriptors of ‘no pain’ (score = 0) and ‘worst pain possible’ (score = 10) [38]. This outcome was chosen as it is a core domain in the consensus statement for tendinopathy [35].

#### Secondary outcomes

The following secondary outcomes will be measured:

1. Pain and disability (measured using the Victorian Institute of Sport Assessment—Achilles (VISA-A)) [39].
2. Global Rating of Change Scale (measured using a 15-point Likert scale ranging from a ‘very great deal worse’ to a ‘very great deal better’). This variable will be dichotomized, with ‘effective’ defined as “somewhat better” or above [40];
3. Function (measured using the Lower Extremity Functional Scale (LEFS) [41]);
4. Health-related quality of life (measured using the VAS component of the EuroQol 5 Dimension 5 Level (EQ-5D-5L) questionnaire [42]);
5. Level of physical activity (measured using the International Physical Activity Questionnaire—short form (IPAQ-SF) [43]).

#### Other outcomes

##### Co-interventions

The use of co-interventions to relieve pain at the Achilles tendon will be measured at 4, 8 and 12 weeks via a REDCap survey™. The use of paracetamol rescue medication (number of participants and mean consumption) or any other treatment (e.g. exercise) to manage their Achilles tendon pain.

##### Adverse events

Adverse events from the interventions (such as skin blistering or the occurrence of new pain or injuries in other areas of the foot and body) will be assessed at 4, 8 and 12 weeks via a REDCap survey™. Participants will be asked to document the type of adverse event, the body location, the duration and severity of the event [44]. An open-response type format will also be available for participant responses.

##### Adherence

Adherence will be measured at 4, 8 and 12 weeks via a REDCap survey™. Participants will be asked to provide information regarding the average number of hours per day and number of days they have worn the heel lifts during the preceding 4 weeks.

### Biomechanical assessment

At baseline, a subpopulation of participants who volunteer (*n* = 40) will undergo a biomechanical assessment to evaluate the acute changes in the biomechanical function of the Achilles tendon as this is the proposed mechanism of action of heel lifts. The variables of interest are Achilles tendon load (ATL), ankle joint moments and plantar/dorsiflexion range of motion (ROM), stance phase duration (SPD), ground reaction force (GRF) and tibial acceleration. One researcher (JB) will perform the assessment, with the support of experts in biomechanical analysis (ST, AG).

After completing a standardised and progressive 7-min warm-up, participants will be asked to walk (5 min) and run (3.5 min) with and without their allocated intervention (i.e. heel lift or sham heel lift) in four testing conditions: (i) walking, shoe only; (ii) walking with the allocated intervention; (iii) running, shoe only and (iv) running with the allocated intervention. The footwear used in testing will be standardised, using the same zero drop Merrell® shoe. The testing sequence will be randomised by a single author (JB). The gait conditions will be fixed at the participant’s optimal walking and preferred running speed to minimise the effect on biomechanical parameters [45].

Optimal walking speed (*V*) will be calculated to be a fourth of maximal walking speed, using the following calculation: *V* = sqrt(*L* × *g* × 0.25) [46]. The formula is based on the Froude number (0.25), leg length (*L*) and acceleration of gravity (*g*). Preferred running speed will be determined using a previously outlined method [47, 48]. Speed will be increased in increments of 0.1 km/h until they first report they have exceeded their preferred ‘comfortable’ running pace. Speed will then be decremented by 0.1 km/h until the participant has confirmed that their ‘comfortable’ running speed has been passed going down. This procedure will be repeated three times and speeds will be averaged to determine the preferred running speed. In between testing, participants will be given as long as required to recover and withdraw if pain exceeds 5 out of 10 on a VAS at any time during the trials [31].

#### Direct measurement of biomechanical variables

Participants will walk and run on an instrumented treadmill (Advanced Mechanical Technology Inc., Watertown, MA, USA) that collects GRF at a sampling rate of 1000 Hz. The grade of the contact surface of the treadmill will be maintained in a horizontal position (0%) throughout testing. Three-dimensional kinematic data will be recorded at a sampling rate of 200 Hz from a 14-camera VICON B-10 system (Oxford Metrics Ltd., Oxford, UK). A biomechanical model will be reconstructed from 38 retroreflective markers, placed on proper landmarks of body segments (Additional file 3), which our research team has used previously [49]. Two inertial measurement units (IMUs) will be placed and aligned with the left and right shank respectively to collect tibial acceleration data. Kinematic, IMU and ground reaction force data will be synchronised and collected through Nexus 2.15 software (Vicon Motion Systems Ltd., Oxford, UK).

#### Achilles tendon load—computed measurement

Achilles tendon load (ATL) will be determined using a method described in similar trials [50–52]. Achilles tendon forces will be estimated by dividing the ankle plantar flexion moment (PFM) by the Achilles tendon moment arm (MA). The MA will be calculated to 1° increments of ankle flexion (*θ*) using the following formula [53], which was derived from magnetic resonance imaging [54]: MA =  − 0.5910 + 0.08297 × *θ* − 0.0002606 × *θ*^2^. The PFM will be computed around the ankle flexion/extension axis (the axis connecting the medial and lateral malleoli) using Newton–Euler inverse dynamics approach and normalised to body mass (kg) [49].

### Sample size

The sample size has been determined a priori using G*Power [55] based on pain intensity at its worst in the previous week as the primary outcome measure. We calculated the sample size using an *f*-test, analysis of covariance (ANCOVA). Using an allocation ratio of 1:1, 90% power, minimal clinically important difference of 1.5 units out of 10 [56], standard deviation of 2.3 [14], moderate effect size [57] (*f* = 0.33) and a significance level set at *a* < 0.05, we estimate that a total of 108 participants will be required (99 and an extra 9 for 10% drop-out) [58].

### Data monitoring

Data will be accessible to the trial investigators (JB, SM, AG, ST, PM) and will be stored on a password-protected computer (JB) and uploaded to LabArchives, a secure cloud-based server. This study will have a Trial Management Committee that will be comprised of study investigators (JB, SM, AG, ST, PM). The Committee will meet every 4 weeks to review safety reports, data quality, protocol adherence and retention rates. An independent Data Monitoring and Ethics Committee was considered to not be needed for this trial, as it is relatively short and includes two safe and common interventions [14]. In addition, the participants are not considered to be vulnerable [59, 60]. There will be no formal interim analysis.

### Statistical analysis

Statistical analysis will be performed using the most recent version of SPSS (IBM Corp., Armonk, NY, USA) available at the time of analysis. Data will be double entered to minimise errors. Demographic data and anthropometric characteristics (e.g. gender, age, foot posture using the FPI, etc.) will be reported by the treatment arm.

The intention-to-treat principle will be used for all participants [61]. Multiple imputation will be used to replace missing data for the primary and secondary outcome measures [62], with the exception being ‘Global Rating of Change Scale, where no data substitution will be applied. Standard tests to assess continuous data for normal distribution will be used and transformation performed if required. The primary outcome measure assessed will be pain intensity (at its worst) at 12 weeks. Continuously scored outcome measures (pain intensity (NRS-11), VISA-A, LEFS, EuroQol 5—VAS, IPAQ-SF and biomechanical outcomes) will be analysed using an ANCOVA with adjustment for baseline scores [63].

Dichotomous data (Global Rating of Change Scale, adverse events and use of co-interventions) will be compared using relative risk, absolute risk increase and number needed to treat or harm. Intervention adherence and CEQ data will be compared between groups using independent *t*-tests. To complement point estimates, standard deviations, 95% confidence intervals and *p*-values will be calculated where appropriate.

## Discussion

Mid-portion AT is common and disabling and up to 40% of people with AT fail to respond to first-line conservative treatment [64]. Heel lifts have recently been found to be more effective than eccentric calf exercise (a guideline recommended treatment) in pain, disability and health-related quality of life outcome measures [14]. However, to our knowledge, there has been no trial comparing heel lifts to a sham [12, 14, 65]. Without the comparison to a sham intervention, it is unknown whether the efficacy of heel lifts is due to specific treatment effects, natural history or placebo. In addition, there are currently no Achilles tendinopathy-specific study investigating heel lifts and their suspected mechanism (i.e. changing biomechanical variables) [16].

The study protocol described here will overcome these limitations. We have included a sham intervention, and our biomechanical assessment is on a tendinopathy-specific cohort. At present, there are no empirically proven guidelines for the prescription of heel lifts (e.g. height or material). Considering this limitation, we chose heel lifts that are supported by the literature [14] and are commercially available to ensure our findings are generalisable to clinical practice. We have deliberately not included exercise, as it may confound the results by reducing symptoms of pain and disability instead of the heel lifts [66]. Instead, we will provide guideline and expert recommended education to both groups [7], which will overcome any ethical concerns of ‘withholding usual care’.

Outcomes from our clinical trial will provide much needed evidence regarding a common biomechanical intervention currently used to manage symptoms in people with mid-portion Achilles tendinopathy. This information will assist clinicians treating people with this condition and may be used in future clinical guidelines.

## Trial status

This is the first version of this protocol (20 July 2023). Advertising for participants commenced in July 2023 and is expected to conclude December 2024. The final results are expected to be available in August 2025 and disseminated via peer review publication and conference presentations.

### Supplementary Information

Supplementary Material 1.

Supplementary Material 2.

Supplementary Material 3.

Supplementary Material 4.

## Data Availability

Data will be made available on request at the completion of the trial.

## Abbreviations

VISA-A: Victorian Institute of Sport Assessment—Achilles
AT: Achilles tendinopathy
SPIRIT: Standard Protocol Items: Recommendations for Interventional Trials
CONSORT: Consolidated Standards of Reporting Trials
NHMRC: National Health and Medical Research Council
FPI: Foot Posture Index
CEQ: Credibility/Expectancy Questionnaire
NRS: Numerical rating scale
LEFS: Lower Extremity Functional Scale
EQ-5D-5L: EuroQol 5D-5L
IPAQ-SF: International Physical Activity Questionnaire—Short Form
ATL: Achilles tendon load
ROM: Range of motion
SPD: Stance phase duration
GRF: Ground reaction force
IMU: Inertial measurement unit
VAS: Visual analogue scale
PFM: Plantarflexion moment
MA: Moment arm
ANCOVA: Analysis of covariance
