# Supplementary material for: Efficacy of heel lifts for mid-portion Achilles tendinopathy (the LIFT trial): study protocol for a randomised controlled trial
Source: Trials. 2024 May 24;25:345. doi: 10.1186/s13063-024-08185-8 (PMC11127406; doi:10.1186/s13063-024-08185-8)
Supplement: Supplementary file 2 — Supplementary Material 2. [file 13063_2024_8185_MOESM2_ESM.docx]

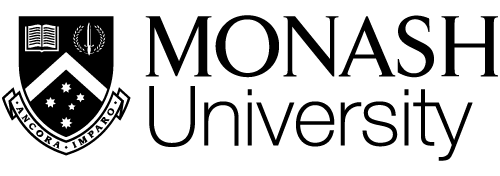


**CONSENT FORM**

**EFFICACY OF SHOE INSERTS FOR MID-PORTION ACHILLES TENDINOPATHY: A RANDOMISED CONTROLLED TRIAL**

Monash University Human Ethics application reference number 36420

*Investigators:*

| Jaryd Bourke  Prof Peter Malliaras | Prof Shannon Munteanu  Dr Alessandro Garofolini  Dr Simon Taylor |
| --- | --- |

I ______________________________ have read and understood the **participant information statement and consent form,** and any questions I have asked have been answered to my satisfaction.

I understand that even though I agree to be involved in this project, I can withdraw from the study at any time, and can withdraw my data up to four weeks following the completion of my participation in the research. Further, in withdrawing from the study, I can request that no information from my involvement be used. I agree that research data provided by me or with my permission during the project may be included in a thesis, presented at conferences and published in journals on the condition that neither my name nor any other identifying information is used.

I consent to being a participant in this research project (please tick relevant box):

ロ NO ロ YES

I also consent to being contacted for future research projects (please tick relevant box):

ロ NO ロ YES

Name of participant (block letters):______________________________________________

Signature:____________________________________________Date:_________________

Name of investigator (block letters):_____________________________________________

Signature:____________________________________________Date:_________________


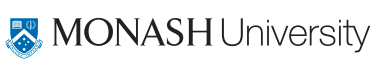


Faculty of Medicine, Nursing and Health Sciences
Monash University
Victoria 3800 Australia

**T** (03) 9904 4502)

**PARTICIPANT INFORMATION STATEMENT**

**EFFICACY OF SHOE INSERTS FOR MID-PORTION ACHILLES TENDINOPATHY: A RANDOMISED CONTROLLED TRIAL**

*Investigators:*

| **Jaryd Bourke**  School of Physiotherapy  Monash University | **Prof Peter Malliaras**  School of Physiotherapy  Monash University | **Prof Shannon Munteanu**  School of Allied Health  Latrobe University |
| --- | --- | --- |
| **Dr Alessandro Garofolini**  Department of Biomechanics  Victoria University | **Dr Simon Taylor**  Department of Biomechanics  Victoria University |  |

**AIM**

This is a student research project aiming to determine the effectiveness of shoe inserts combined with education for the treatment of mid-portion Achilles tendinopathy.

**FUNDING**

This study is funded by the Australian Podiatry Education and Research Foundation.

**RATIONALE**

Achilles tendinopathy is common and painful. Although shoe inserts are regularly used for this condition, the effectiveness and the mechanism of the treatment is unknown.

**TEST PROCEDURE**

**You can participate in this study if you:**

i) are aged between 18 to 65 years;

ii) have had pain in your Achilles tendon for at least 6 weeks;

iii) in the last week had pain in your Achilles tendon greater than 3 out of 10 (if 0 = no pain and 10 = worst imaginable pain);

iv) own footwear that can fit the shoe inserts and be willing to use them for at least 8 hours per day;

v) are willing to attend Victoria University (Melbourne, Australia) once for an assessment;

vi) are willing to attempt to not receive additional treatments (such as physical therapy, foot orthoses, shoe modifications, intra-articular injections, or surgery) for your Achilles tendon pain during the course of the study (12 weeks);

vii) are willing to attempt to discontinue taking all pain-relieving medications to relieve pain at your Achilles tendon(s) (except paracetamol) for at least 14 days prior to baseline assessment and during the study period (12 weeks);

**You will not be able to participate in this study if you:**

i) are pregnant;

ii) had previous surgery on your Achilles tendon(s);

iii) ruptured your Achilles tendon(s);

iv) have any other painful conditions within the foot or ankle that are more painful than your Achilles tendon pain;

v) have an injury (e.g., severe ankle instability) or medical condition (e.g., type 2 diabetes) that makes you unsuitable for inclusion;

vi) currently have inflammatory arthritis (e.g. rheumatoid arthritis and gout) and/or neurological disorders;

vii) treatment with shoe inserts for your Achilles tendon pain in the previous 3 months

viii) taken fluoroquinolones within the previous 2 years;

ix) history of breast cancer and/or use of oestrogen inhibitors

x) are unable to speak or read English

xi) Have received any injections (such as corticosteroids) at the Achilles tendon(s) or surrounding area in the previous 3 months

Investigators will enquire about the above inclusion/exclusion criteria over the phone and at the initial appointment.

**STUDY DURATION AND FOLLOW-UP APPOINTMENTS**

The effects of the treatment on the pain and disability caused by your Achilles tendinopathy will be assessed over 12 weeks.

As a study participant, your involvement will be as follows:

Initial assessment: We will send you a survey to collect your baseline data and assess the pain in your Achilles tendon. Then we will arrange an appointment with you at Victoria University, Footscray where we will determine your suitability to be in the study, provide you with your treatments, and if you are happy to volunteer, a biomechanical assessment of how you walk with and without your shoe inserts (90-150 minutes).

Electronic surveys (every 4 weeks): We will assess your adherence, use of other treatments and if any adverse events have occurred (survey will take 10-20 minutes to complete).

Week 12 assessment: Electronic survey where we will reassess the pain in your Achilles tendon(s) (survey will take 10-20 minutes).

Each of these appointments will now be discussed below in more detail.

**Initial assessment**

To confirm the presence of mid-portion Achilles tendinopathy and assess how you walk and run, an initial assessment will be required. This assessment is expected to take a maximum of 2.5 hours and will involve a clinical assessment and an ultrasound scan of your Achilles tendon at Victoria University, Footscray.

Once the assessments are complete, the investigator will inform you of your eligibility to participate in this study. If you are eligible, you will be invited to participate in this study and will be randomly allocated to one of two different shoe insert treatments (see section titled “Treatments” below). This means you have a 50:50 chance of receiving either shoe insert. You will not have a choice as to which insert you receive.

The following will also be assessed using questionnaires:

1. the types of pain you are experiencing in your Achilles tendon(s);
2. your ability to perform everyday tasks;
3. your general health-related quality of life;
4. physical activity levels;
5. your beliefs and expectations about the treatment provided.

Next, if you are happy to volunteer, we will evaluate how the treatment allocated to you influences how you walk and run. Declining to be a part of the biomechanical testing will not affect your participation in this study. If you consent to participate in the biomechanical testing, we will attach reflective markers (similar to small table tennis balls) to your legs and feet with adhesive tape. We will then record you walking and running on a treadmill. There are infrared cameras positioned to detect the movement of the reflective markers, and from this, a computer program can create an image of dots on a black screen that we can use to measure how you walk and run. We will also attach inertial measurement units (small devices, the same size as a match box) to measure the stresses in your shin bone.

These tests are non-invasive and should not cause you discomfort. You will be attached to a safety harness to minimise your risk of injury. If you experience increased pain in your Achilles tendon(s) (any pain greater than a 5 out of 10) or a new area of pain, testing will be stopped. Some of the reflective markers will be positioned on your chest and back, which can be obscured by clothing. If your clothes during testing obscures the markers, we may ask to 1) adjust your clothing with clips; 2) for you to wear a t-shirt that we will provide; and/or 3) perform the testing without a t-shirt.

**Electronic survey (every 4 weeks)**

You will be emailed a survey link every 4 weeks, which you will need to complete. If you prefer a postal survey, this can be arranged. We will be assessing:

i) your use of any other treatments to relieve pain in your Achilles tendon(s);

ii) your adherence with the treatments;

iii) any adverse reaction to the shoe inserts.

**Week 12 survey**

Twelve weeks after the initial assessment, you will be emailed another survey link. If you prefer a postal survey, this can be arranged. We will be assessing:

i) the amount of pain and disability caused by the Achilles tendon condition;

ii) the magnitude of improvement since you have started using your shoe inserts.

iii) your general health-related quality of life;

iv) your physical activity levels;

v) your use of any other treatments to relieve pain in your Achilles tendon(s);

vi) your adherence with the treatments;

vii) any adverse reaction to the shoe inserts.

**TREATMENTS**

**Shoe inserts**

Because two treatments are being compared in this study, you will be randomly allocated to receive one of two types of shoe insert treatments.

You will be given one pair to use on both sides (left and right) and will be required to wear your allocated shoe inserts every day for at least 8 hours for the study duration (12 weeks). You will be provided with an information sheet that will provide instructions for adjusting to and using the shoe inserts.

Once the study is completed, you will be offered the alternative treatment if it is shown to be a more effective treatment than the one you received.

**Education**

At the initial appointment, you will also receive education on acceptable pain levels. The information will be included in the information sheet as a reminder, along with the instructions on how to use your shoe inserts.

**USE OF PAIN-RELIEVING MEDICATIONS AND OTHER FORMS OF TREATMENT DURING THE STUDY PERIOD**

As a participant in this study we recommend that you use paracetamol (Panadol®), up to 4 grams/day, as a pain-relieving medication if it is necessary during the study period (12 weeks). You must attempt to not use any other treatment for your Achilles tendon pain during the study period. However, if you do not obtain sufficient pain relief with this approach, you are free to use other treatments or take other medication as you require. It is possible that limiting the amount of (or altering) pain medication or treatment may cause an increase in pain in your Achilles tendon(s).

**EXPECTED BENEFITS OF BEING IN THIS STUDY**

As a study participant, you will receive a thorough diagnosis of the pain in your Achilles tendon, education about your tendon pain and one of two types of shoe inserts. We cannot guarantee that these treatments will result in an improvement in your Achilles tendon pain. Your involvement in this research study will assist health-care practitioners in choosing the best treatment(s) for people affected by Achilles tendon pain.

**RISKS AND DISCOMFORTS**

**Shoe inserts**

The shoe inserts prescribed in this study are commonly used by podiatrists and physiotherapists. You will be supplied with a brochure that will detail how to use the inserts. The occurrence of serious harmful effects from wearing the shoe inserts is extremely rare. However, you may experience mild discomfort in the feet, legs and even your back during the initial stages (first two weeks) of wearing your inserts. However, this discomfort will resolve. If you do experience some discomfort when adjusting to your inserts, you may want to apply a cold pack (wrapped in a damp towel) on the affected area for 10-15 minutes, 3-4 times per day.

If any of these effects fail to reduce despite the following advice above or if you have any concerns, you are advised to contact a study investigator (Jaryd Bourke). Jaryd will arrange a consultation for you at Monash University, Clayton to advise you on how to deal with the harmful effect(s) and determine the need for further referral, if any.

**Paracetamol**

Paracetamol is a common medication used for mild to moderate pain and can be purchased at a supermarket without a prescription. Common side effects of using paracetamol include nausea, fatigue, and rashes. However, the occurrence of any side effect is rare when taken at the correct dose. There is no obligation for you to use paracetamol and if you have any concerns, we encourage you to contact your General Practitioner (GP) or pharmacist to enquire about your suitability to use this medication. If you feel that you are experiencing any side effects from the paracetamol you will need to stop taking the medication and visit your GP or nearby hospital as required.

**COSTS TO PARTICIPANTS**

As a participant of this study, you do not have to pay for the treatment you receive. If you attend the initial assessment and are deemed ineligible for the study, there will also be no cost to you. You will need to pay for all travel and parking fees to attend your appointment at Victoria University.

**ENQUIRIES**

Any questions regarding this project may be directed to the Primary Investigator (Jaryd Bourke).  **FREEDOM OF CONSENT**

Participation in this project is entirely voluntary. You are free to deny consent before or during the study. In the latter case, such withdrawal of consent should be performed at any time you specify. Your participation and / or withdrawal of consent will not influence your present or future involvement at Monash University. In the case that you are a student at Monash University, it will not influence your grades or progress in your course of study. Your consent form will be uploaded to a secure cloud-based server and then shredded. No one apart from the investigators listed in the beginning of this participant information statement will have access to the computer records and written records. The raw data and uploaded consent forms will be stored in a password protected computer of one of the researchers and destroyed in five years’ time after collection using secure methods.

You have the right to withdraw from active participation in this project at any time and, further, to demand that data arising from your participation are not used in the research project provided that this right is exercised within four weeks of the completion of your participation in the project. You are asked to complete the “Withdrawal of Consent Form” or to notify the investigator by email or telephone that you wish to withdraw your consent for your data to be used in this research project.

**PUBLICATION AND OTHER USE OF RESULTS**

It is possible that results from this study will be displayed in a postgraduate thesis, presented at a conference, or published in a peer review journal. Data from the study may also be submitted to a data repository that is owned by a scientific journal. This means that other researchers may be able to access the data.

A number of measurements will be compared between the initial appointment and the follow-up appointment. This will include measurements of pain in the Achilles tendon(s), use of pain relieving medications and amount of unpleasant effects. It should be noted, however, that participant information will be expressed anonymously (e.g., participant 1, participant 2, etc.), with no mention of your name or personal details. The results of the study and your individual results will be made available to you upon your request. This may entail mailing of results to your home residence, emailing to your preferred email address, or if you prefer, a discussion with Jaryd Bourke in person.

The results obtained from this study may also be used in future research projects. Such projects may include studies investigating shoe inserts and other musculoskeletal conditions, such as heel pain. The investigators listed at the beginning of this participant information statement will have access to your data. In instances where other researchers need to access your data for future research projects, the Monash University Human Ethics Committee will be advised and requested to grant permission to do so. If you consent, we may also contact you in the future to enquire about your availability to participate in other research projects.

**COUNSELING SERVICES**

If at any time you feel distressed by the trial, the following links to mental health services are available:

Beyond Blue: <https://www.beyondblue.org.au/>

Black Dog Institute: <https://www.blackdoginstitute.org.au/>

**COMPLAINTS**

If you have any complaints that the investigator has not been able to answer to your satisfaction, you may contact the Monash Human Research Ethics Committee at managerresearchethics@monash.edu. Please quote the application reference number 36420.
