# Supplementary material for: Efficacy of heel lifts for mid-portion Achilles tendinopathy (the LIFT trial): study protocol for a randomised controlled trial
Source: Trials. 2024 May 24;25:345. doi: 10.1186/s13063-024-08185-8 (PMC11127406; doi:10.1186/s13063-024-08185-8)
Supplement: Supplementary file 3 — Supplementary Material 3. [file 13063_2024_8185_MOESM3_ESM.docx]

**Additional file 2:** Biomechanical model

A set of 38 retroreflective markers arranged in a cluster setup will be used to track 3D position of body segments. Each cluster will comprise of a group of individual markers that represent a single body segment (e.g., shank). This includes left and right foot clusters (10 markers), left shank cluster (4 markers), right shank cluster (4 markers), left thigh cluster (4 markers), right thigh cluster (4 markers), pelvis cluster (4 markers), trunk cluster (4 markers) and head cluster (4 markers). To minimise effects of skin movement artefact^1,2^, we will secure the semirigid clusters over extra-long neoprene bands made of anti-migration material that are wrapped and fastened on the thigh and shank segments, and a headband used for the head. Individual trunk and pelvis retroreflective markers will be placed over the 7^th^ cervical vertebrae, 10^th^ thoracic vertebrae, sterno-clavicular notch, xiphoid process, and posterior- and anterior-superior iliac spines. The markers for the feet segments have been screwed into the heel, lateral 1^st^ and 5^th^ metatarsal head, tip of the 1^st^ toe and top of the 2^nd^ metatarsal head of the Merrell footwear, ensuring repeatable marker location between ‘shoe-insert’ and ‘no shoe-insert’ conditions.

Before capturing the dynamic trials, a static pose (1 second) will be recorded where an additional 10 retroreflective markers will be placed on anatomical landmarks (e.g., lateral malleoli) identified by palpation. The static pose will be used to calibrate the position and orientation of the upper and lower body skeletal system (excluding arms). A dynamic calibration will be captured after the participants walk 2-3 steps during their warmup. Post-testing, marker gaps <10 frames will be filled via the spline fill and any >10 frames will be filled with a combination of pattern, rigid body, or cyclic fill technique. Raw data will be exported to Visual 3D (C-motion) and filtered using a low-pass Butterworth filter (4th order, zero lag) with a cut-off frequency of 15 Hz^3^. A six-degrees of freedom segment model will be built for biomechanical analysis.. For joint rotations, we will use right-handed orthogonal coordinate systems, where the z-axis represents the axial direction of the segment. The x-axis lied in the frontal plane perpendicular to the z-axis. The y-axis lied on the sagittal plane in the antero-posterior direction. In Visual3D, joint angles will be calculated using an x–y–z Cardan–Euler sequence representing flexion/extension, abduction/adduction and axial rotation of the head, trunk, thigh, shank and foot^4^. For the pelvis, the Cardan sequence will be reversed (z–y–x), as recommended by Baker^5^. Joint angles will be normalized to the subject static reference position, recorded as a “standing calibration trial”. For the scope of this study, the segment movements of interest will be those during stance phase and within the sagittal plane only (i.e., flexion/extension rotations). Stance phase will be defined as any time there is >20N of vertical force applied to the force platform^3^. The force signal recorded will be assigned to the relevant foot segment based on detection software in Visual3D. The estimated foot assigned to the force will be based on the proximity between the location of the centre of mass of the foot and the transverse plane location of the centre of pressure on the force plate. Force signals will then be used to compute joint moments (through inverse dynamic calculations) represented in the joint coordinate system and normalised to body mass (kg)^6^.

**References**

1. Taylor W, Ehrig R, Duda G, Schell H, Seebeck P, Heller M. On the influence of soft tissue coverage in the determination of bone kinematics using skin markers. J Orthop Res. 2005;23:726–34.
2. Leardini A, Chiari L, Della Croce U, Cappozzo A. Human movement analysis using stereophotogrammetry: Part 3. Soft tissue artifact assessment and compensation. Gait & Posture. 2005;21:212–225.
3. Garofolini A, Oppici L, Taylor S. A real-time feedback method to reduce loading rate during running: effect of combining direct and indirect feedback. J Sports Sci. 2020;38:2446-53.
4. Robertson D, Robertson G, Caldwell G, Hamill J, Kamen P, Whittlesey S. Research Methods in Biomech, 2^nd^ ed.; Human Kinetics: Champaign, IL, USA, 2013.
5. Baker R. Pelvic angles: A mathematically rigorous definition which is consistent with a conventional clinical understanding of the terms. Gait & Posture. 2001;13:1–6.
6. Schache A, Baker R. On the expression of joint moments during gait. Gait & Posture. 2007;25:440–52.
