# Supplementary material for: Efficacy of heel lifts for mid-portion Achilles tendinopathy (the LIFT trial): study protocol for a randomised controlled trial
Source: Trials. 2024 May 24;25:345. doi: 10.1186/s13063-024-08185-8 (PMC11127406; doi:10.1186/s13063-024-08185-8)
Supplement: Supplementary file 4 — Supplementary Material 4. [file 13063_2024_8185_MOESM4_ESM.docx]

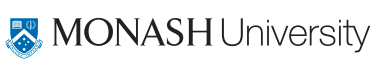


Faculty of Medicine, Nursing and Health Sciences
Monash University
Victoria 3800 Australia

**T** (03) 9904 4502)

**INSTRUCTIONS ON USING YOUR SHOE INSERTS**

We have given you a pair of **shoe inserts** to wear for at least **8 hours per day**.

Your Achilles tendon pain will generally be while you are **weight-bearing**. Therefore, it is important you use your **shoe inserts** while **walking**, **running** and/or any other **weight-bearing activities e.g., cycling**.

To use, **first** remove the insole or molded footbed from your shoe. **Second,** place the shoe insert we provided you with in your shoe (navy side up). If you wear multiple types of footwear (e.g., work and sport shoes), you will need to transfer the **shoe inserts** into all the shoes you use during the day.

**Discomfort**It is rare, but some people may experience mild discomfort in their feet, legs and even their back while adapting to their shoe inserts (1-2 weeks).

This discomfort will resolve. If you do experience some discomfort while adjusting to your shoe inserts, you may want to apply a cold pack (wrapped in a damp towel) on the affected area for 10-15 minutes, 3-4 times per day.

If the discomfort persists after 2 weeks or you find the shoe inserts painful (extremely rare), please contact us immediately and we will arrange a consultation for you at Monash University, Clayton to advise you and determine the need for further referral, if any.

**Pain levels**During the study, we would like you to continue with your regular jobs and activities/hobbies (e.g., sport). However, we ask that you keep your Achilles tendon pain levels below a 5 out of 10. If **ten (10) = the worst pain imaginable** and **zero (0) = no pain.**

Your pain is allowed to reach a 5 out 10 during activity but should have subsided back to a 0 by the following morning. During activity, if your Achilles tendon pain exceeds a 5 out of 10, please reduce your activity/exercise (if possible).

**Contact**Thank you for participating in this study. Your input will assist future health-care practitioners in choosing the best treatment(s) for people with Achilles tendinopathy.

**If you have any questions or concerns, you are advised to contact the Primary Investigator of this study, Jaryd Bourke.**
